# AI in the Shadows: Unveiling the Strengths and Blind Spots of Medios AI Retinal Screening in Cancer Care

**DOI:** 10.7759/cureus.99002

**Published:** 2025-12-11

**Authors:** Deepsekhar Das, Bhavna Chawla, Neiwete Lomi, Sumit Grover, Atindra Narayan

**Affiliations:** 1 Ophthalmology, All India Institute of Medical Sciences, New Delhi, IND; 2 Medicine, National Cancer Institute, All India Institute of Medical Sciences, New Delhi, IND

**Keywords:** age related macular degeneration, ai, diabetic retinopathy, glaucoma, leukemic retinopathy, medios ai

## Abstract

Introduction

Artificial intelligence (AI) is increasingly being integrated into ophthalmic diagnostics, offering potential for efficient screening of common retinal diseases. The Medios AI system by Remidio (Singapore, Singapore), designed for use with a smartphone-based fundus camera, claims to detect diabetic retinopathy (DR), age-related macular degeneration (ARMD), and glaucoma. However, its performance in complex clinical settings such as ocular oncology remains underexplored. This study aims to evaluate both the diagnostic capabilities and limitations of the Medios AI system when applied to a diverse cohort of oncology patients.

Materials and methods

An observational study was conducted over three months in an ocular oncology clinic. Ninety-eight cancer patients (196 eyes) underwent fundus photography using the Remidio smartphone-based fundus imaging system. The images were analyzed using the Medios AI algorithm. AI-generated findings were compared with clinical evaluations by an experienced ophthalmologist to identify diagnostic concordance and discrepancies. Additional attention was paid to the system’s imaging capabilities, including its ability to capture wide-field or montage images.

Result

The AI system accurately identified glaucomatous cupping in three patients, flagged two cases of DR, and detected signs of ARMD in two patients-all consistent with clinical examination. However, eight patients with leukemic retinopathy were incorrectly flagged as having DR, revealing a lack of specificity in distinguishing vascular retinal pathologies. The system also failed to detect optic atrophy, a critical neuro-ophthalmic finding in oncology patients. A technical limitation was also noted: the inability of the Remidio system to generate montage or wide-field images, restricting visualization of the peripheral retina.

Conclusion

While the Medios AI system demonstrates promise in identifying common retinal pathologies such as DR, ARMD, and glaucoma, its limitations are significant in the oncology context. The inability to distinguish similar hemorrhagic retinopathies, failure to detect optic nerve atrophy, and lack of wide-field imaging capabilities underscore the need for cautious implementation. Integration of AI tools must be accompanied by expert clinical oversight, especially in specialized settings where retinal presentations are complex and atypical.

## Introduction

Artificial intelligence (AI) is revolutionizing medical diagnostics by introducing tools that promise faster, more accessible, and often cost-effective solutions for disease detection. Ophthalmology has emerged as a particularly fertile ground for AI applications, owing to the visually intensive nature of diagnostics and the high burden of preventable vision loss worldwide. Among the retinal diseases most commonly screened using AI tools are diabetic retinopathy (DR), age-related macular degeneration (ARMD), and glaucoma, conditions that collectively contribute to a significant proportion of global blindness [1-3].

The Medios AI system, developed by Remidio, integrates a deep learning algorithm with a smartphone-based fundus camera, aiming to provide point-of-care diagnostic support, especially in low-resource settings. Its utility has been demonstrated in primary eye care and diabetic screening programs. However, its robustness in more complex and nuanced clinical environments remains uncertain. One such domain is ocular oncology, where retinal findings often extend beyond the common pathologies targeted by the AI and include atypical or mixed presentations [4-6].

Ocular oncology involves the diagnosis and management of intraocular tumors and paraneoplastic syndromes, as well as systemic malignancies with ocular manifestations. In such patients, retinal findings can include leukemic retinopathy, choroidal metastases, optic nerve atrophy, and hemorrhagic retinopathies - all of which may be subtle, varied, or overlapping in their presentation. An AI system that lacks the nuance to distinguish these can yield misleading results, potentially compromising patient care if used without appropriate clinical oversight.

This study evaluates the diagnostic accuracy and limitations of the Medios AI system (Medios Technologies, Singapore, Singapore), which is a clinically validated, CE-marked artificial intelligence software running on the Remidio Non-Mydriatic Fundus On Phone, in a real-world oncology setting. By comparing AI-generated findings with assessments made by a trained ophthalmologist, we aim to identify both the capabilities and shortcomings of the AI tool. Additionally, we assess the technical performance of the smartphone-based imaging system itself, particularly its ability or lack thereof to capture wide-field and montage images, which are critical for comprehensive retinal assessment in oncology patients.

## Materials and methods

Study design and setting

A retrospective cross-sectional observational study was carried out over a period of three months at the National Cancer Institute, All India Institute of Medical Sciences, New Delhi. The aim was to assess the diagnostic performance of the Medios AI system in a specialized cohort of patients with known or suspected malignancies. All patients presenting to the Ophthalmology Outpatient Department were included in the study. Informed consent was obtained from all participants.

Participants

A total of 98 patients (196 eyes), all diagnosed with systemic malignancies, were enrolled in the study. These included patients undergoing active treatment for leukemia, lymphoma, and various tumors with ocular complaints.

Patients presenting to the Ophthalmology Outpatient Department at the National Cancer Institute, AIIMS, New Delhi, with any form of cancer or history of cancer, and who gave their consent, were included in the study. Patients with corneal ulcers, significant cataract, vitreous hemorrhage where images of fundus could not be captured, and uncooperative, moribund patients where positioning of the fundus camera equipment was not feasible were excluded from the study.

Imaging procedure

Fundus images were captured using the Remidio smartphone-based fundus camera (Remidio Innovative Solutions, Singapore, Singapore). The imaging was performed by Ophthalmologists under standardized lighting conditions. Both macular and optic disc regions were imaged, with attempts made to capture the peripheral retina where possible. However, the system’s technical capacity to obtain montage or wide-field images was inherently limited.

AI analysis

The images were analyzed using the Medios AI system, which is pre-configured to detect signs of DR, ARMD, and glaucomatous optic neuropathy. The AI provides an output label along with confidence scores for each diagnosis.

Clinical evaluation

Simultaneously, all patients underwent a comprehensive ophthalmic examination by the ophthalmologist. This included slit-lamp biomicroscopy, indirect ophthalmoscopy, and additional diagnostic tests as necessary.

Outcome measures

The primary outcome was diagnostic concordance between AI-generated findings and clinical diagnoses. Secondary outcomes included identification of false positives and false negatives, analysis of misclassifications, and evaluation of the technical limitations of the imaging system itself. SPSS software (IBM Corp., Armonk, NY) was used for the analysis of data.

## Results

Demographics and patient characteristics

Of the 98 patients included in the study, 54 were male, and 44 were female, with a mean age of 52.3 years (range: 8-78 years). The underlying malignancies included leukemia (23 patients), lymphoma (10 patients), and various tumors such as mandibular carcinoma, Eyelid carcinoma, Breast carcinoma, lung carcinoma, gall bladder carcinoma, glioblastoma, medulloblastoma, pituitary macroadenoma, and melanoma (65 patients).

AI diagnostic concordance

Glaucoma

**Figure 1:**
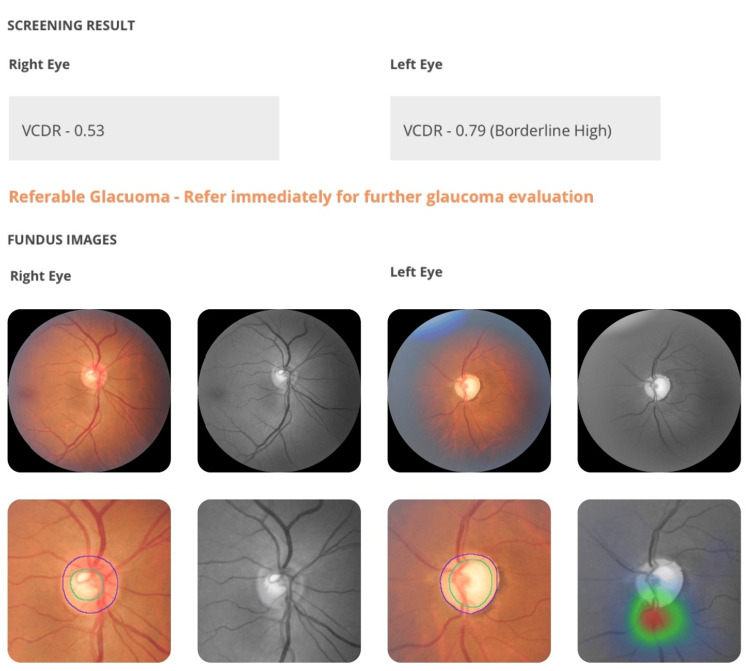
Correct diagnosis of glaucomatous cupping with inferior neuroretinal rim thinning flagged by AI (green and red circles) in left eye of a patient with breast carcinoma VCDR: Vertical Cup Disc Ratio

The AI correctly identified glaucomatous cupping in three patients, with findings corroborated by clinical examination and confirmed by visual field testing and OCT. These patients had optic disc cupping ratios greater than 0.7 and neuroretinal rim thinning (Figure 1).

Diabetic Retinopathy

**Figure 2:**
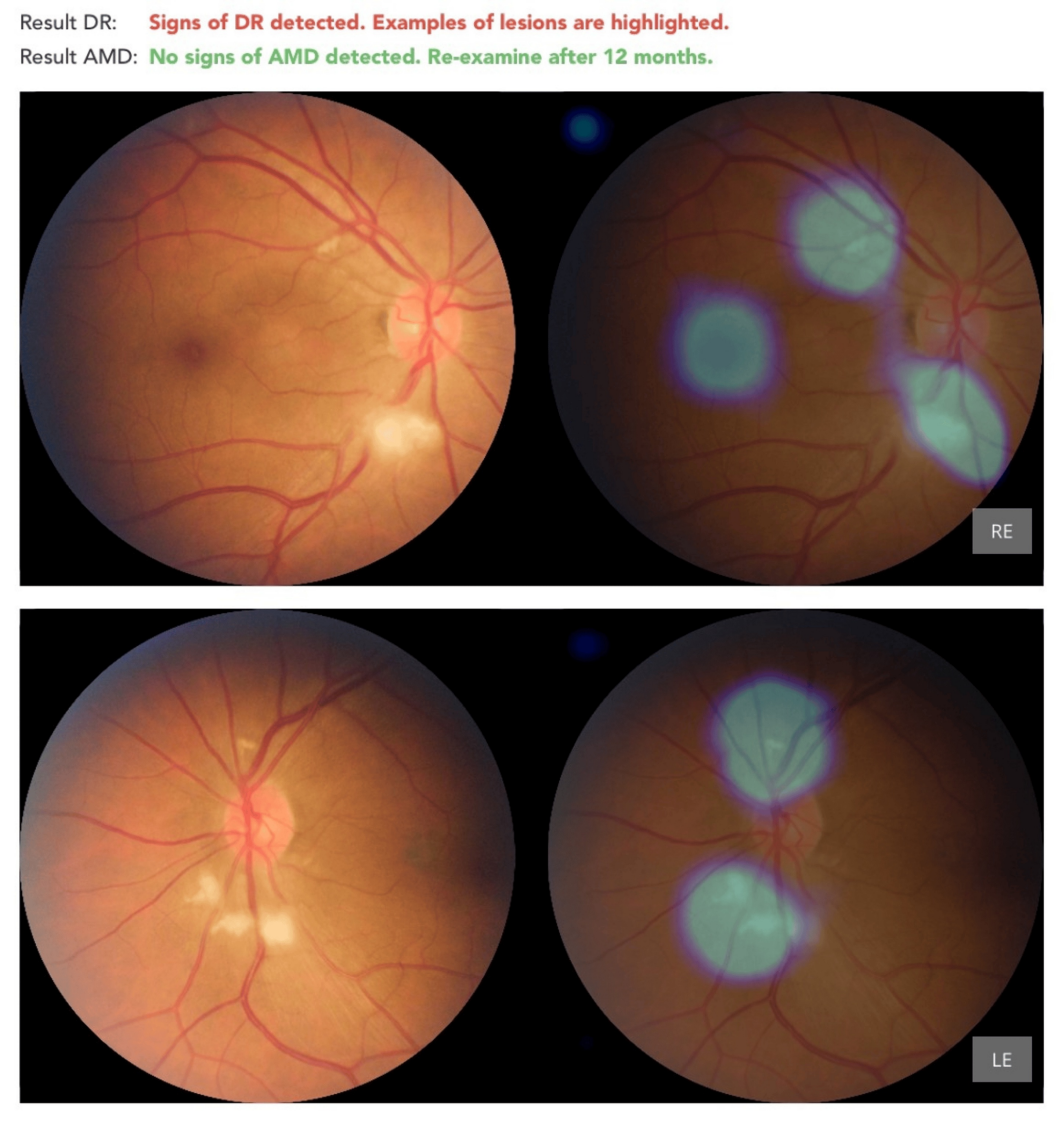
Correct diagnosis of diabetic retinopathy in a patient with gall bladder carcinoma Presence of hard exudates is flagged by AI as sky blue circles. AMD: Age-Related Macular Degeneration; DR: Diabetic Retinopathy

**Figure 3:**
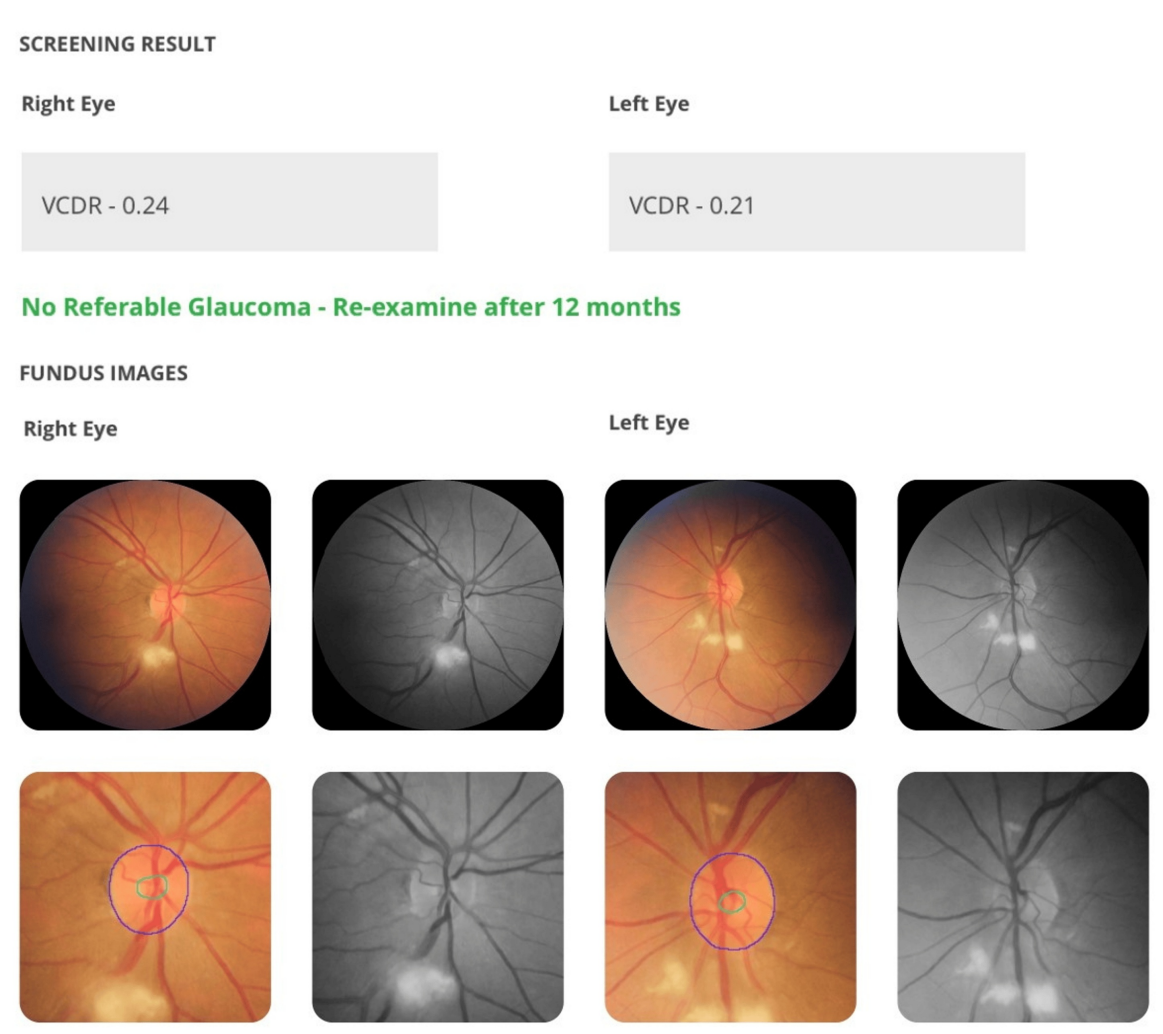
Correct diagnosis of diabetic retinopathy in a patient with lung carcinoma Presence of retinal hemorrhages is flagged by AI as sky blue circles. AMD: Age-Related Macular Degeneration; DR: Diabetic Retinopathy

The system flagged 10 cases of DR. Upon clinical review, only two patients were confirmed to have true DR. Notably, eight of these were leukemia patients with leukemic retinopathy, which presents with similar retinal hemorrhages, cotton wool spots, and venous changes. This suggests a significant issue with specificity (Figure 2 and Figure 3).

Age-Related Macular Degeneration

**Figure 4:**
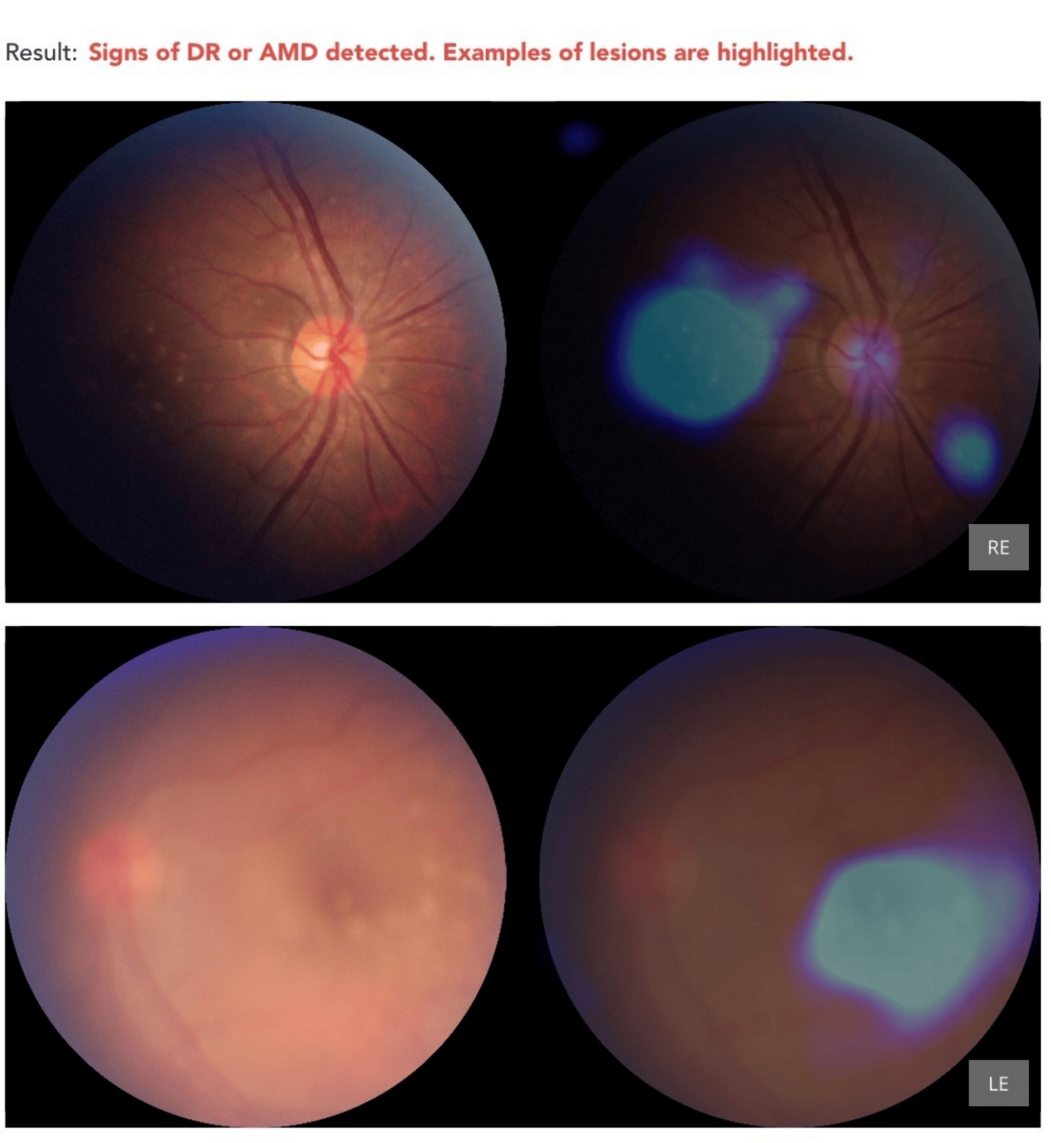
Correct diagnosis of ARMD with visible drusens in a patient with breast carcinoma Presence of Drusens is flagged by AI as sky blue circles. ARMD: Age-Related Macular Degeneration

The AI successfully detected ARMD in two patients, both of whom had early to intermediate dry ARMD. The findings included drusen and retinal pigment epithelium (RPE) irregularities (Figure 4).

Missed diagnoses and limitations

Leukemic Retinopathy

**Figure 5:**
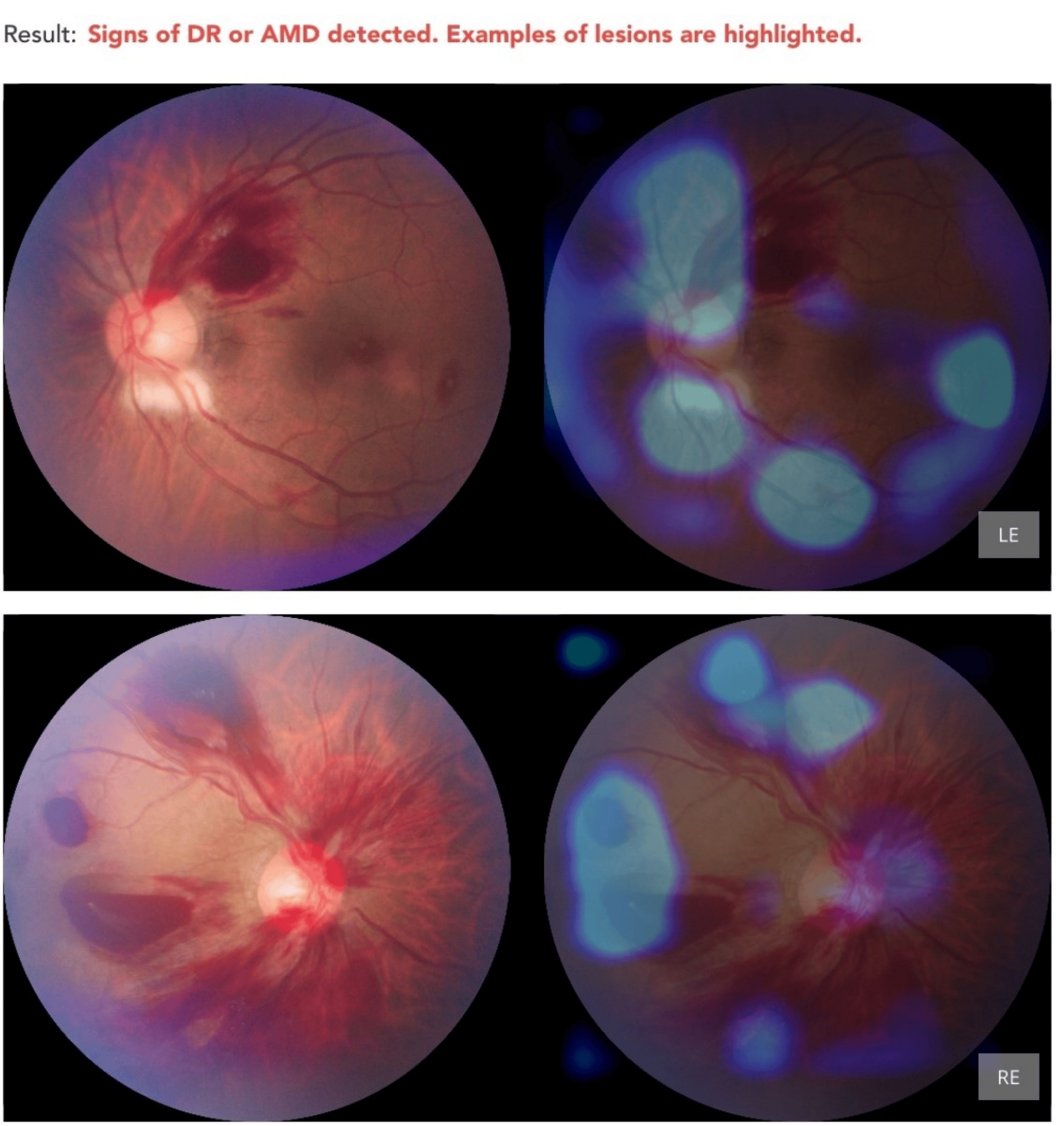
Incorrect diagnosis of DR or AMD in a patient with bilateral leukemic retinopathy with acute myeloid leukemia AMD: Age-Related Macular Degeneration; DR: Diabetic Retinopathy; LE: Left Eye; RE: Right Eye

**Figure 6:**
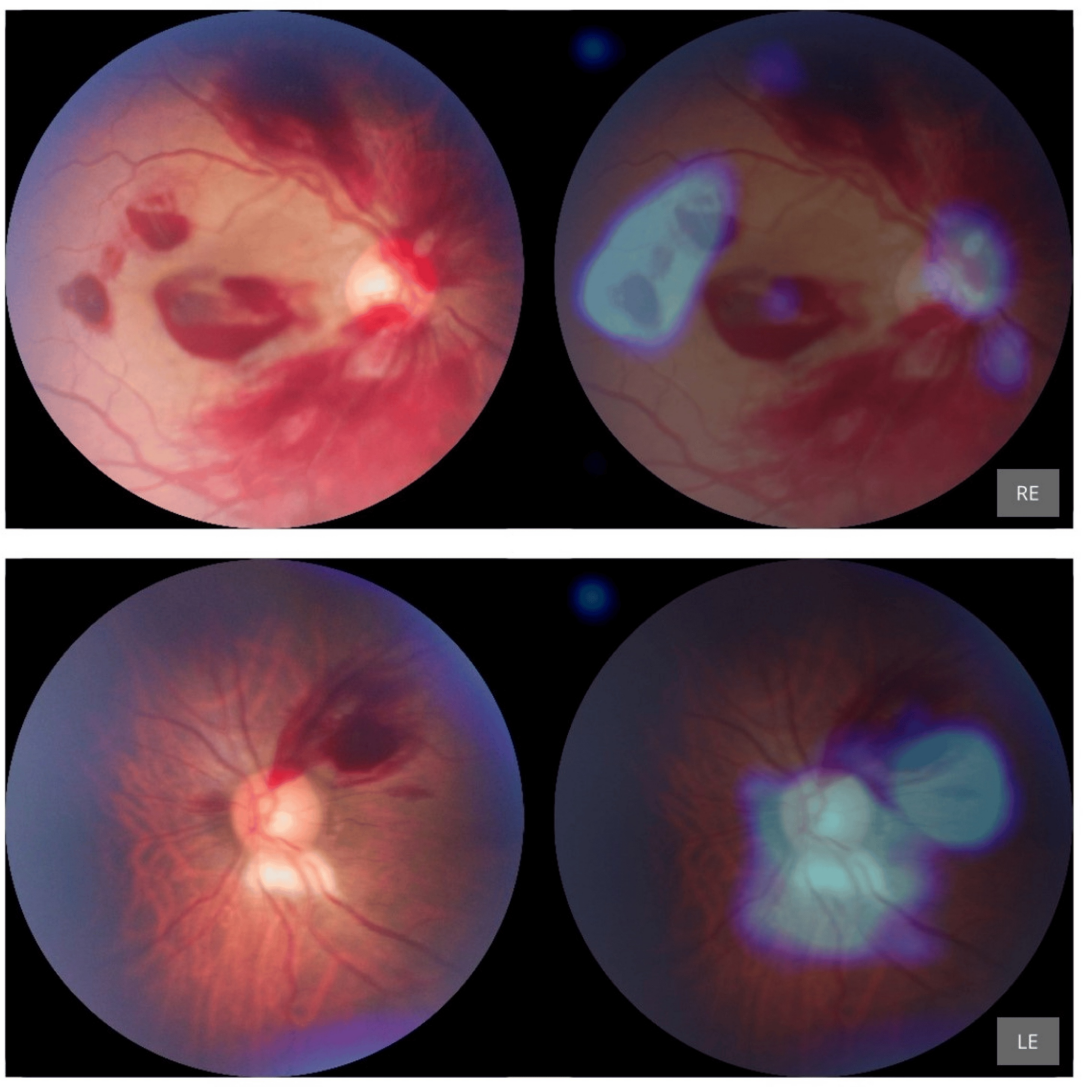
Incorrect diagnosis of DR in a patient with leukemic retinopathy with acute myeloid leukemia Presence of retinal hemorrhages is flagged by AI as sky blue circles. DR: Diabetic Retinopathy

None of the eight cases of leukemic retinopathy were accurately classified by the AI. Instead, these were all misclassified as DR, highlighting the AI’s current limitation in differentiating between vascular retinopathies with overlapping features (Figure 5 and Figure 6).

Optic Atrophy

**Figure 7:**
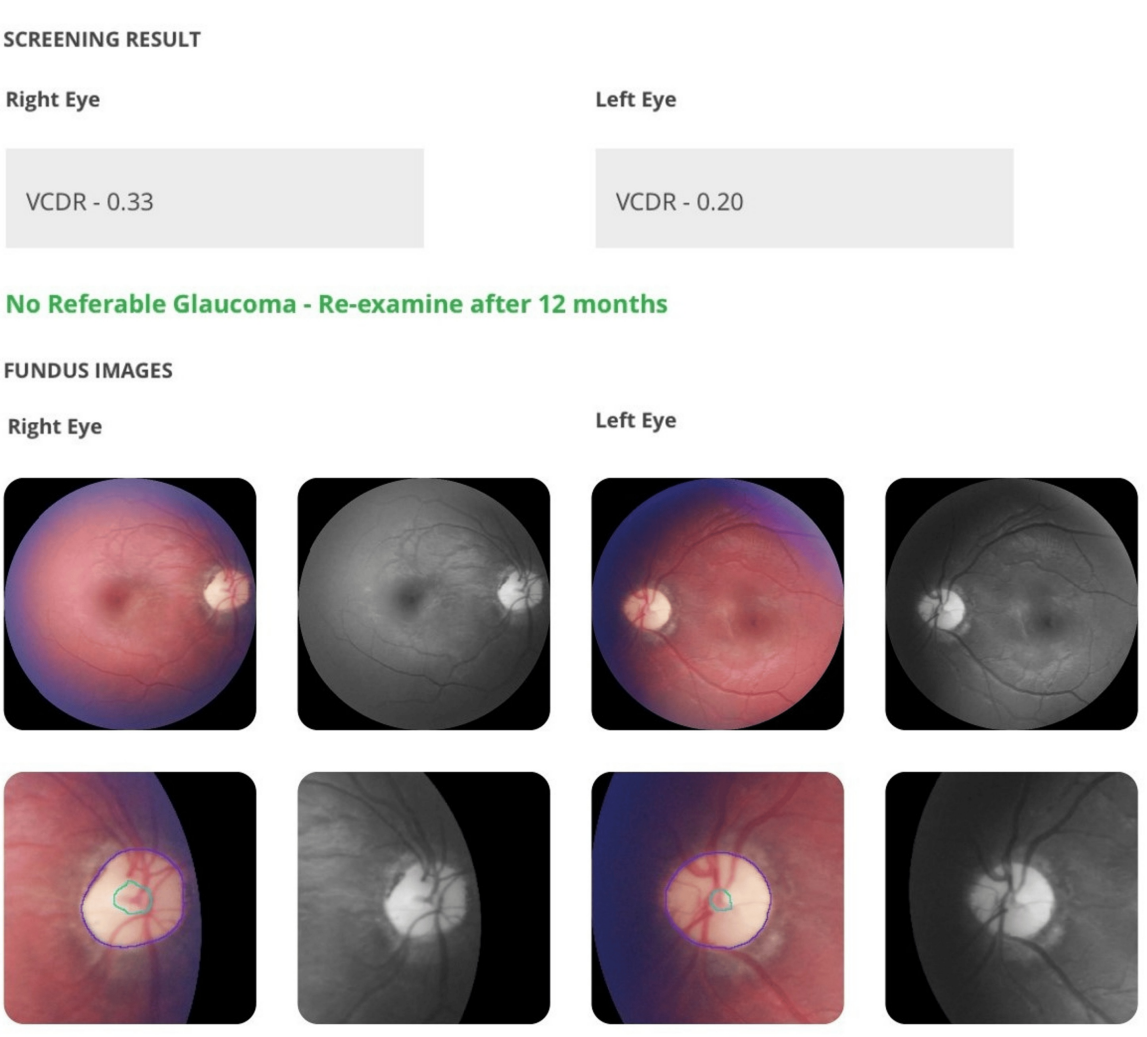
Inability to detect optic atrophy in a patient with optic atrophy with medulloblastoma Evident pale optic disc is not flagged by AI.

The AI failed to detect optic atrophy in five patients. These findings were clear on clinical examination, such as pallor of the optic disc. This is particularly concerning, given the significance of optic nerve pathology in oncology patients with possible central nervous system involvement or paraneoplastic syndromes (Figure 7).

The images flagged and missed by AI were correlated with clinically confirmed diagnoses, and missed/misclassified findings were tabulated (Table 1).

**Table 1:** Diagnostic performance of Medios AI system

Retinal Condition	Cases Detected by AI	Confirmed Clinically	Correct Diagnoses	Misclassified/Missed Findings	% Missed
Glaucoma (Cupping)	3	3	3	None	0
Diabetic Retinopathy	10	2	2	8 cases of leukemic retinopathy misclassified	80%
Age-Related Macular Degeneration (ARMD)	2	2	2	None	0
Leukemic Retinopathy	0 (all misclassified)	8	0	All 8 labelled as DR by AI	100%
Optic Atrophy	0	5	0	5 completely missed	100%
Peripheral Retinal Lesions (e.g., Metastases, Infiltrates)	Not detectable (due to hardware)	Several noted clinically	0	Missed due to lack of wide-field/montage	NA

Technical limitations of the imaging system

The Remidio system does not support montage or wide-field imaging. There was one patient with DR involving the periphery, which was missed by the requisite scans required for AI assessment by Medios. Peripheral retinal pathology commonly seen in choroidal metastasis and retinal infiltrates was frequently missed due to this constraint. The field of view was insufficient in several cases to assess the full extent of pathology, limiting both AI and clinician interpretation.

## Discussion

The integration of AI into ophthalmology has shown considerable promise, particularly in the creation of informed consents, procuring data regarding ophthalmology, and the detection of common retinal diseases such as DR, ARMD, and glaucoma [7,8]. The Medios AI system, when used in primary eye care settings, has demonstrated high sensitivity and specificity. For instance, Natarajan et al. [1] reported a sensitivity of 100% and specificity of 88.4% for referable DR using Medios AI in a community-based screening program. Similarly, the SMART study [2] found a sensitivity of 93% and specificity of 95.5% for detecting any stage of DR using the same platform.

These findings are consistent with broader trends in the field. For example, Gulshan et al. [3], using a deep learning model developed by Google, achieved a sensitivity of 90.1% and specificity of 98.2% for DR detection across multiethnic populations. Moreover, the IDx-DR system, approved by the FDA, demonstrated a sensitivity of 87% and specificity of 90% in a pivotal clinical trial [9]. Other systems have also been shown to effectively detect a spectrum of retinal diseases, including glaucoma and ARMD, using high-resolution fundus photographs [10,11].

However, most of these studies have focused on generalized or screening populations. Our current study highlights the challenges posed when these AI systems are applied to a specialized cohort, such as oncology patients, where retinal findings often deviate from typical presentations. The Medios AI system showed limitations in accurately identifying leukemic retinopathy, frequently misclassifying it as DR. This is not entirely surprising, as leukemic retinopathy often presents with similar features - retinal hemorrhages, venous dilation, and cotton wool spots - that may be interpreted by the AI as diabetic changes [12,13].

Equally concerning was the AI system's failure to detect optic atrophy in oncology patients. Optic nerve pallor, which may arise from compressive lesions, CNS metastases, or paraneoplastic syndromes, is a critical finding that demands urgent attention [14]. The inability of the AI system to identify such neuro-ophthalmic features is a significant shortcoming, especially given that other AI algorithms have begun to show early promise in identifying optic neuropathies from fundus photographs [15].

Beyond diagnostic misclassification, our findings also reveal technical constraints inherent in the imaging hardware. The Remidio smartphone-based fundus camera lacks the capacity to capture wide-field or montage images. This limitation hinders the assessment of peripheral retina, where many oncology-related lesions, such as choroidal metastases and peripheral retinal infiltrates, may be located [8]. While the central retina is sufficiently imaged for basic DR or ARMD screening, the constrained field of view restricts the utility of both clinician and AI interpretation in oncology practice.

These findings highlight a broader issue in digital health: the generalizability of AI models. Most existing systems are trained on standard datasets dominated by common pathologies and clear imaging conditions. Applying these systems to outlier populations without appropriate calibration or validation can yield misleading results. Interdisciplinary collaboration between AI developers, ophthalmologists, and healthcare policymakers will be key to developing ethically robust, clinically useful, and culturally contextualized AI tools [8].

## Conclusions

The Medios AI system, in conjunction with the Remidio smartphone-based fundus camera, demonstrates strong potential for detecting common retinal pathologies in primary or screening settings. However, this utility is markedly reduced in complex clinical environments such as ocular oncology. The system’s tendency to misclassify leukemic retinopathy as DR, failure to detect optic atrophy, and inability to capture peripheral retinal lesions due to hardware constraints underscore its current limitations.

These findings suggest that AI tools, while valuable, must be integrated into clinical workflows with caution, particularly in specialized settings. Future developments should focus on expanding training datasets to include diverse and atypical retinal presentations, improving specificity in pattern recognition, and enhancing imaging hardware capabilities. Until such advancements are realized, expert clinical oversight remains indispensable for accurate diagnosis and safe implementation.
